# Adaptive sparse attention-based compact transformer for object tracking

**DOI:** 10.1038/s41598-024-63028-5

**Published:** 2024-05-28

**Authors:** Fei Pan, Lianyu Zhao, Chenglin Wang

**Affiliations:** 1 School of Computer Science and Engineering, Tianjin University of Technology, Liqizhaung street, Tianjin, 300384 China; 2 School of Mechanical Engineering, Tianjin University of Technology, Liqizhaung street, Tianjin, 300384 China

**Keywords:** Object tracking, Siamese network, Transformer, Adaptive sparse attention, Computational science, Computer science, Information technology

## Abstract

The Transformer-based Siamese networks have excelled in the field of object tracking. Nevertheless, a notable limitation persists in their reliance on ResNet as backbone, which lacks the capacity to effectively capture global information and exhibits constraints in feature representation. Furthermore, these trackers struggle to effectively attend to target-relevant information within the search region using multi-head self-attention (MSA). Additionally, they are prone to robustness challenges during online tracking and tend to exhibit significant model complexity. To address these limitations, We propose a novel tracker named ASACTT, which includes a backbone network, feature fusion network and prediction head. First, we improve the Swin-Transformer-Tiny to enhance its global information extraction capabilities. Second, we propose an adaptive sparse attention (ASA) to focus on target-specific details within the search region. Third, we leverage position encoding and historical candidate data to develop a dynamic template updater (DTU), which ensures the preservation of the initial frame’s integrity while gracefully adapting to variations in the target’s appearance. Finally, we optimize the network model to maintain accuracy while minimizing complexity. To verify the effectiveness of our proposed tracker, ASACTT, experiments on five benchmark datasets demonstrated that the proposed tracker was highly comparable to other state-of-the-art methods. Notably, in the GOT-10K^1^ evaluation, our tracker achieved an outstanding success score of 75.3% at 36 FPS, significantly surpassing other trackers with comparable model parameters.

## Introduction

Object tracking is an important research task in the field of computer vision, utilizing initial target state information to determine the target’s state in each video frame. the preceding frames’ state information serves as a cornerstone for predicting the subsequent state, with prolonged tracking sessions prone to accumulating errors across sequences, potentially jeopardizing tracking efficacy. Furthermore, obstacles like target deformation, scale fluctuations, and occlusions pose additional challenges. In pursuit of heightened tracking precision, researchers have invested significant efforts, employing methods such as designing Siamese network frameworks that effectively transforms target tracking into an end-to-end learning process focused on gauging the similarity between the target image and the search region^2^, and employing methods like precise target bounding boxes^3^.

However, a significant proportion of the aforementioned methods predominantly rely on cross-correlation operations to assess the degree of similarity between the target template and the search region, which may fall into local optima^4^. In recent years, with the research and development of Transformers, the shortcomings of previous trackers in handling long sequences have been addressed, showcasing the advantages of Transformer-based object tracking methods built upon Siamese networks^4–9^. Despite their usefulness, these trackers exhibit several noteworthy shortcomings. Firstly, a common limitation among them is their reliance on ResNet as backbone. This backbone lacks the prowess to efficiently grasp global information and is constrained in its feature representation abilities. Consequently, it struggles significantly when confronted with occlusion and similarity issues, often falling short in effectively addressing such complexities. Secondly, they overutilize multi-head self-attention (MSA) focus on global information, while often misses crucial local details, particularly the target information within the search region. This oversight blurs the demarcation between the foreground and background. Despite the utilization of Sparse Attention in sparsett^8^, there remains an issue of inflexibility stemming from the fixed threshold, which renders the sparse attention insufficiently adaptive. Thirdly, these trackers struggle to harness historical trajectory information to address challenges like motion occlusion and similar targets. Lastly, these trackers often rely on bulky models in pursuit of marginal accuracy gains, which not only complicates the deployment process but also imposes significant strain on the operational environment, resulting in the limited applications of Transformer-based object trackers. Therefore, how to solve aforementioned these challenges is a worthwhile research question.

In this work, we consider how to efficiently grasp global information and improve feature representation capability. At the same time, on the basis of sparse attention, solve the problem of fixed threshold and poor scalability. Moreover, we need to improve the robustness of online tracking and streamline the intricacies of the tracker model. To solve these problems, we designed a new method (ASACTT) that contains three key components: Swin-Transformer-based feature extraction, adaptive sparse attention-based feature fusion, and prediction head. The Swin-Transformer-based feature extraction is improved Swin-Transformer-Tiny, which can extract global information and improve feature representation capability, thereby adeptly addressing challenges like occlusion and interference from similar objects. The adaptive sparse attention-based feature fusion comprises multi-head attention and ASA, which can improve the Transformer’s focusing ability on target-relevant information within the search region. Unlike traditional sparse attention, the ASA can adaptive adjust the sparse threshold based on the length of the input image sequence and the complexity of the images. Finally, the prediction head can regress the prediction box and locate the tracking object. During the tracking process, our DTU integrate position encoding and historical candidate information, ensuring the purity of the initial frame while adeptly adapting according to the changes in the appearance of the target.

In summary, the main contributions of this work are as follows:

1. We improve Swin-Transformer-Tiny as the backbone for the tracker, efficiently grasping global information and improve feature representation capability.
2. We propose an ASA effectively solves the problem of fixed sparse attention threshold and poor scalability.
3. We design a novel DTU, combined with position encoding and historical candidate information, ensuring the purity of the initial frame representation while adeptly adapting to changes in the target’s appearance.
4. Extensive experiments demonstrate the superiority of our approach across five benchmark datasets, while running at 36 FPS, providing evidence for the effectiveness of our method.

## Related work

### Siamese in object tracking

Based on the Siamese network tracker, the renowned SiamFC^2^ transforms the task of object tracking into an end-to-end learning problem of similarity between the target image and the search region. This breakthrough surpasses the limitations of pre-trained Convolutional Neural Networks (CNNs) and reveals general relationships between object motion and appearance, such as naive cross-correlation^2^, deep cross-correlation^10^, pixel-wise cross-correlation^3^, etc. However, cross-correlation involves a local linear matching process that can easily fall into local optima^4^. Furthermore, despite their capabilities, most of Siamese network trackers still face difficulties in handling complex scenarios such as target deformation, partial occlusion and changes in viewpoint. This impediment arises from the cross-correlation process, which while capturing relationships, can disrupt the semantic integrity of input features, ultimately hindering the precise perception of target boundaries.

### Transformer in object tracking

With the outstanding performance of Transformer in handling long sequences and large models, a plethora of tracking methods based on Transformer have emerged. DETR^6^ transforms object tracking into a bounding box prediction problem, while STARK^11^ adapts the Transformer architecture of DETR to address the issue. In this approach, the encoder processes global spatio-temporal feature information between the target and search region and the decoder predicts the target’s positional information, achieving commendable results.

TrDiMP^5^ building upon DiMP^11^, combines Siamese networks and Transformer by using a CNN backbone, encoder and Transformer decoder. In this context, Transformer is employed to enhance the target template and search region. Similar to previous Siamese trackers, TrDiMP utilizes cross-correlation to measure the similarity between the target template and the search region, which may impede high-performance tracking. Addressing this drawback, TransT^4^ replace cross-correlation with Transformer to fuse features. ToMP^7^ further extends the model predictor, allowing for the estimation of precise bounding box regression weights. SwinTrack^12^ adopts the Swin-Transformer as backbone, further enhancing the tracker’s feature extraction capability. Due to the ability of Transformer-based trackers to integrate richer semantic information, they exhibit higher accuracy compared to Siamese network-based trackers.

### Other interesting works in visual tracking

In the realm of visual tracking, certain works have stood out due to their focus on refining the attention mechanisms in Transformers^8,9,13–17^. Most of these trackers excel in managing lengthy sequential relationships and capturing comprehensive global information, yet they tend to overlook target-specific details within the designated search region. Although a few methods^8,9,13^ focus on local details, there are problems such as complex calculations or inflexible parameter thresholds. Therefore, based on traditional sparse attention, we adaptive adjust the sparse threshold according to the length of the input image sequence and the complexity of the image.

Several noteworthy research endeavors and effective plugins have contributed to the advancement of trackers, including NeighborTrack^18^ and Alpha-Refine^3^. NeighborTrack, employs a unique method where it pre-trains on a Re-ID network specific to certain categories and simultaneously tracks multiple similar targets. By utilizing bipartite matching and neighbor information to determine target-candidate frame compatibility, it effectively reduces tracking errors. Our DTU is deeply inspired by this innovative methodology. Moreover, Alpha-Refine utilizes pixel-wise correlation, corner prediction heads and auxiliary mask heads to extract maximum spatial information for precise bounding box estimation. Drawing inspiration from their concepts, we have adapted and redefined our target prediction bounding boxes, achieving enhanced precision in tracking.

## Proposed method

**Figure 1:**
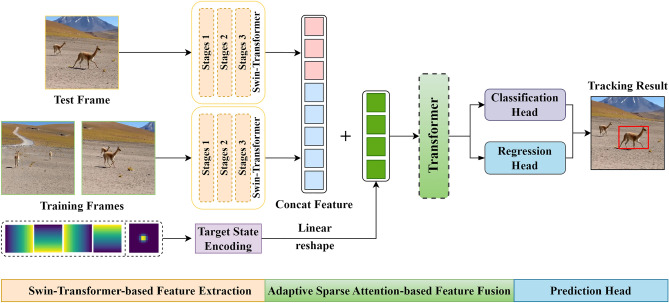
The network architecture of ASACTT. It consists of three key components: Swin-Transformer-based feature extraction, adaptive sparse attention-based feature fusion, and prediction head. Despite its remarkably compact model parameters (only 21.9M), our ASACTT demonstrates excellent performance.

We propose a novel tracker named ASACTT based on the Siamese network framework, as illustrated in Fig. 1. It primarily consists of three parts: Swin-Transformer-based feature extraction, adaptive sparse attention-based feature fusion and prediction head. Firstly, we introduce the feature extraction and target encoding based on Swin-Transformer-Tiny in Sect. "Swin-transformer-based feature extraction”. Secondly, we describe the feature fusion network based on ASA (including attention mechanism and position encoding) in Sect. "Adaptive sparse attention-based feature fusion”. Finally, in Sect. "Head and training loss", we present a detailed overview of our prediction head network, which includes both classification and regression heads. Subsequently, we will delve into the key components of the tracking network.

### Swin-transformer-based feature extraction

Currently, Resnet is widely adopted as the backbone for most object trackers^3–7,9,10,19–21^, owing to its exceptional feature representation capabilities and robust generalization properties. However, it falls short in capturing the hierarchical and local characteristics of images, as well as in efficiently handling large-scale image data computation. Therefore, we utilize Swin-Transformer as a feature extractor to address these limitations. Swin-Transformer not only excels in extracting target features but also assists subsequent networks in providing more precise location information.

Although our tracker ASACTT is based on the Siamese network framework, different from most trackers, we use a discriminative tracking model similar to ToMP to locate the target object in the test frames. As opposed to generative models, our approach offers more flexibility in classification boundaries, higher accuracy and faster training speed. The weights of the prediction model are obtained using a model optimizer, requiring a pair of image patches as input: one extracted from the training frame patch $\text {x}\in {{\mathbb {R}}^{{{H}_{x}}\times {{W}_{x}}\times 3}}$ and the other from the test frame patch $\text {y}\in {{\mathbb {R}}^{{{H}_{y}}\times {{W}_{y}}\times 3}}$. Here, $x\in \frac{{{H}_{x}}}{s}\frac{{{W}_{x}}}{s}\times C$ encapsulates the training image features, while $\text {y}\in \frac{{{H}_{y}}}{s}\frac{{{W}_{y}}}{s}\times C$ represents the testing image features. The variable *s* signifies the backbone’s stride, and *C* denotes the dimension of the image features. To optimize model parameters and enhance performance, we have strategically reduced the original backbone’s feature dimension from 384 to 256, taking into account the entire model framework.

To enhance the performance of the target model and derive more refined weights, we postulate that for each training frame ${{F}_{train}}\in \left\{ \left( {{q}_{i}},{{p}_{i}} \right) \right\} _{i=1}^{m}$, the predicted state of the target model is ${{p}_{i}}\in \gamma$. Here, ${{q}_{i}}\in \chi$ represents the deep feature map extracted from the training images, and *m* signifies the total count of training frames. The optimization function that guides our search for the optimal weights is formulated as follows:

1 $$\begin{aligned} \omega =\underset{\varpi }{\mathop {\arg \min }}\,\sum \limits _{\left( q,p \right) \in {{F}_{train}}}{f\left( h\left( \varpi ;q \right) ,p \right) }+\lambda g(\varpi ), \end{aligned}$$

where the objective function comprises the residual function *f*, which quantifies the discrepancy between the target model’s output $h\left( \varpi ; q \right)$ and the ground truth label *p*. The regularization term $g(\varpi )$ ensures the smoothness and generalizability of the model, weighted by the scalar $\lambda$. The variable $\varpi$ represents the optimal weights that we seek to determine for the target model. Within the training frame set ${{F}_{train}}$, we leverage the tracker’s predictions as pseudo-labels, which serve as valuable supervisory signals. By minimizing the objective function through this optimization process, we ensure that the predictive model attains weights that are optimally calibrated, thus enhancing its ability to effectively discriminate between the target of interest and its surrounding background.

### Adaptive sparse attention-based feature fusion

**Figure 2:**
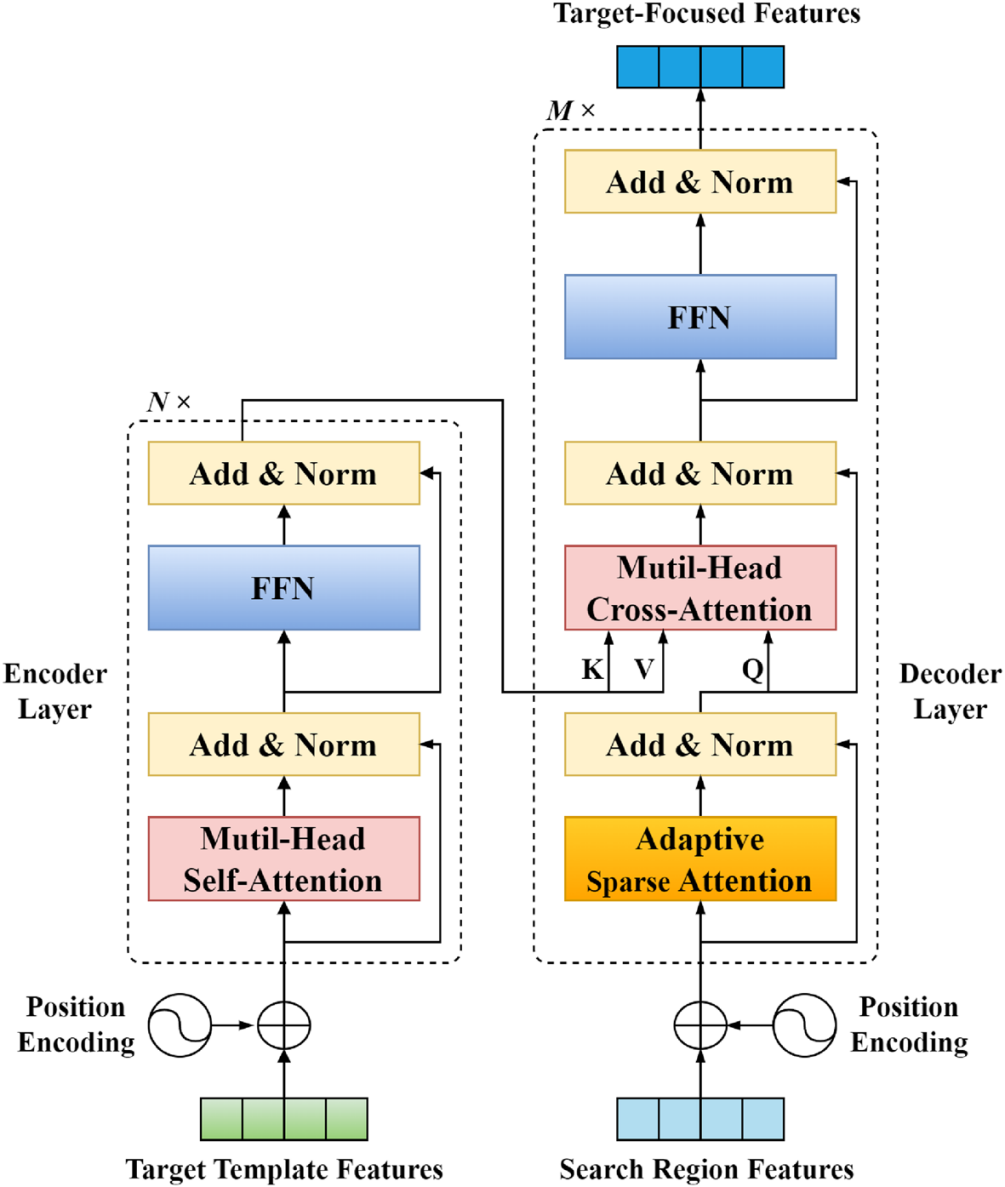
The architecture of adaptive sparse attention-based feature fusion network. Comprising multiple encoders and decoders, the encoders consist of PE, MSA and FFN, while the decoders consist of ASA, MCA and FFN.

To significantly enhance the tracker’s proficiency in recognizing foreground target objects, we have focused our efforts on the target itself and tackled the crucial issue of target localization. To this end, we have refined a feature fusion network that leverages ASA. This network, built upon the robust Transformer architecture, exhibits an encoder-decoder framework, as depicted in Fig. 2. The encoder serves to encode intricate features of the target template, while the decoder adeptly decodes features from the search region, ultimately culminating in the generation of highly focused target features.

End-to-end learning training is employed to generate model predictions directly from the data, facilitating the seamless integration of target-specific priors into the prediction model. This integration empowers the model to not just discern between the target and observed background characteristics, but also to accentuate the crucial feature information of the target. Additionally, our prediction model fuses the current test frame with previously honed features, leveraging the feature information of the present test frame to predict a more apt target model. Unlike traditional approaches that rely on a static feature space defined by a pre-trained feature extractor, our method dynamically crafts a more discriminative feature space for each frame, maximizing the utilization of target-specific information.

#### Encoder

The feature fusion network encoder consists of multiple blocks, positional encoding (PE), multi-head self-attention (MSA), and a feed-forward network (FFN). This architecture boasts the ability to conduct global reasoning across the entire image, as well as across numerous training and testing features. To begin, we encode the target state information in the training frames, fuse it with depth image features to handle foreground and background information in both training and testing frameworks. Later, a distinct encoding layer identifies testing frames. To boost target localization precision, positional encoding is a key input to the prediction model. This positional data is integrated into the encoder, enhancing its target identification and analysis within the imagery.

**Position Encoding** To gain a deeper and more comprehensive understanding of the target state, we devise a position encoding framework that encompasses two fundamental components: target information encoding and target range encoding. This framework incorporates not just the crucial bounding box (Bbox) details of the target, but also its range and precise positional data. To facilitate this, we leverage *H* and *W*, representing the height and width of the feature map associated with the target template. The target foreground, denoted as ${{e}_{fg}}\in {{\mathbb {R}}^{1\times C}}$, along with the target’s position center, ${{q}_{i}}\in {{\mathbb {R}}^{H\times W\times 1}}$, form the cornerstone of our approach. The target position function is succinctly expressed as:

2 $$\begin{aligned} \psi ({{q}_{i}},{{e}_{fg}})={{q}_{i}}\cdot {{e}_{fg}}. \end{aligned}$$

Drawing inspiration from the established benchmarks for target tracking research, outlined in^7,22^, we initially employ the notation ${{b}_{i}}=\{b_{i}^{x},b_{i}^{y},b_{i}^{w},b_{i}^{h}\}$ to represent the precise location and dimensions of the target object’s bounding box in the training frames labeled *i*. Subsequently, we transform each coordinate point $({{j}^{x}},{{j}^{y}})$ on the depth image features${{x}_{i}}$ into the corresponding position in the image domain, utilizing the mapping function $({{k}^{x}},{{k}^{y}})=(\left\lfloor \frac{s}{2} \right\rfloor +s\cdot {{j}^{x}},\left\lfloor \frac{s}{2} \right\rfloor +s\cdot {{j}^{y}})$. Following this, we calculate the normalized distance for each transformed position, relative to the four defining edges of the bounding box ${{b}_{i}}$. This process is formalized as follows:

3 $$\begin{aligned} \begin{aligned} l_i=(k^x-b_i^y)/W_{im},r_i=(k^x-b_i^x-b_i^w)/W_{im}, \\t_i=(k^y-b_i^y)/H_{im},b_i=(k^y-b_i^y-b_i^h)/H_{im}, \end{aligned} \end{aligned}$$

where ${{W}_{im}}=s\cdot W$and${{H}_{im}}=s\cdot H$ represent the scaled image width and heigh, These normalized distances, serving as the four boundaries, are then utilized to generate a dense set of bounding boxes $d=(l,t,r,b)$, where $d\in {{\mathbb {R}}^{H\times W\times 4}}$. Next, we utilize a Multilayer Perceptron (MLP), denoted as $\phi$, to encode the bounding boxes. This process results in the derivation of position encoding as detailed below:

4 $$\begin{aligned} {{P}_{enc}}={{q}_{i}}\cdot {{e}_{fg}}+\phi ({{d}_{i}}). \end{aligned}$$

Subsequently, we refine the depth image ${{x}_{i}}$ of the target, enhancing its dimensionality from 4 to *C*, which enables us to capture the target’s state information as:

5 $$\begin{aligned} {{X}_{i}}={{x}_{i}}+{{q}_{i}}\cdot {{e}_{fg}}+\phi ({{d}_{i}}). \end{aligned}$$

Here ${{X}_{i}}\in {{\mathbb {R}}^{H\times W\times C}}$ encapsulates the encoded target state information. The resulting fused feature map serves as the input for the encoder. Consequently, the encoder’s operation can be expressed as:

6 $$\begin{aligned} Encoder(X)=\left\{ \begin{matrix} f_{enc}^{i}\left( {{X}_{i}}+{{P}_{enc}} \right) ,\quad i=1 \\ f_{enc}^{i}\left( Z_{enc}^{i-1} \right) ,\quad 2\le i\le N \\ \end{matrix}. \right. \end{aligned}$$

Where $X\in {{\mathbb {R}}^{H\times W\times C}}$ represents the feature of the target template, $f_{enc}^{i}$ denotes the *i*-th encoder layer, and $Z_{enc}^{i-1}\in {{\mathbb {R}}^{H\times W\times C}}$ signifies the output of the ($i-1$)-th encoder layer.

#### Decoder

The decoder of the feature fusion network differs from the encoder. The essence of its uniqueness stems from the sequential processes it undergoes in each layer. Initially, it utilizes ASA (Attention-based Self-Attention) to compute the self-attention of the input *X*, offering a profound understanding of the data’s intrinsic relationships (see details further below). Subsequently, it employs MCA (Multi-head Cross-Attention) to capture the cross-attention between the input *X* and *Y*. The decoder then integrates the encoder’s output features (${{Z}_{i}}$ and ${{Z}_{test}}$) and sums them, serving as input to the Transformer decoder. This integration process closely resembles the operations within a layer of the encoder, albeit with a distinct purpose and context. This can be mathematically expressed as:

7 $$\begin{aligned} Decoder(Y,Z_{enc}^{N})=\left\{ \begin{matrix} f_{dec}^{i}\left( {{Y}_{i}}+{{P}_{dec}},Z_{enc}^{N} \right) ,i=1 \\ f_{dec}^{i}\left( Z_{dec}^{i-1},Z_{enc}^{N} \right) ,2\le i\le M \\ \end{matrix}. \right. \end{aligned}$$

Here, $Y\in {{\mathbb {R}}^{{{H}_{s}}\times {{W}_{s}}\times C}}$ represents the features of the search region, while$Z_{enc}^{N}\in {{\mathbb {R}}^{{{H}_{t}}\times {{W}_{t}}\times C}}$ denotes the template features encoded by the encoder. The function $f_{dec}^{i}$ corresponds to the *i*-th layer of the decoder, and $Z_{dec}^{i-1}\in {{\mathbb {R}}^{{{H}_{s}}{{W}_{s}}\times C}}$ represents the output of the preceding ($i-1$)th decoder layer.

**Adaptive Sparse Attention (ASA)** Distinguishing itself from other sparse attention mechanisms, ASA adaptive tunes the sparse threshold $K_{A}$ in accordance with the input image sequence’s length and the intricacies of its constituent images. This approach empowers the model to selectively concentrate on specific segments of the input sequence, leveraging positional cues. Consequently, the foreground receives a more targeted focus, and its edge regions become more discernible. The complexity assessment of the input is derived from the length of the image sequence *n* and the feature vectors $Y_i\in \mathbb {R}^{H_s\times W_s\times C}$ of each individual image. As a result, the complexity measurement function can be expressed as:

8 $$\begin{aligned} F_\text{C}=\frac{1}{n}\sum _{i=1}^n|\left. Y_i\in \mathbb {R}^{H_s\times W_s\times C}\right| _2, \end{aligned}$$

where *i* denotes the index within the image sequence, $Y=Y_1,Y_2,...,Y_n$ represents the comprehensive feature set of all images in the sequence, and $\left| \cdot \right| _2$ signifies the L2 norm. Once the complexity metric is established, we can deduce an adaptive threshold that aligns with the complexity of the image sequence:

9 $$\begin{aligned} K_A=\alpha \cdot F_C+\beta , \end{aligned}$$

where $\alpha$ and $\beta$ are hyperparameters, $\alpha$ governs the extent to which the output of the complexity measurement function affects the threshold, while $\beta$ provides a baseline threshold. By fine-tuning these parameters, we gain control over the sparsity level of the model.

### Head and training loss

In the preceding section, we delineated the intricate yet efficacious feature fusion network architecture that masterfully pinpoints the core of the target, thereby ensuring the acquisition of highly accurate target bounding boxes. Central to our design is a streamlined prediction head network, composed of two distinct modules: a regression branch that calculates the bounding box for the target’s position, and a classification branch for predicting the category at each position.

**Prediction head network** To mitigate downsampling quantization errors, we predict offset values and normalized bounding box sizes. We enrich the model input by decoding target size along with its central position. Extending the prediction variables, we estimate bounding box regression network weights alongside target model weights. Specifically, we apply a linear layer to the output of the Transformer decoder $Z_{dec}^{i-1}\in {{\mathbb {R}}^{{{H}_{s}}{{W}_{s}}\times C}}$ to obtain weights for bounding box regression ${{w}_{bbreg}}$ and target classification ${{w}_{cls}}$. These derived weights are then directly integrated into the prediction model $h({{w}_{cls}},{{z}_{test}})$ to enhance its predictive capabilities. Furthermore, we leverage these weights to condition the output of the Transformer Encoder’s testing features ${{Z}_{test}}$. This conditioning informs accurate bounding box regression. Finally, by classifying, scoring, and predicting the target box, we identify the highest-confidence candidate, thus boosting tracking performance.

**Loss functions** In terms of classification, we employ a cross-entropy loss method for target classification, and further integrate diverse losses to cater to both background and foreground regions. As for regression, we chose the GIoU loss function, leveraging left-top-right-bottom (ltrb) bounding boxes to supervise the bounding box regression. The comprehensive loss function is articulated as follows:

10 $$\begin{aligned} L={{\lambda }_{cls}}{{L}_{cls}}({\hat{y}},y)+{{\lambda }_{giou}}{{L}_{giou}}({\hat{d}},d). \end{aligned}$$

Here, ${{\lambda }_{cls}}$ and ${{\lambda }_{giou}}$ serve as weighted scalars, each dedicated to balancing the importance of classification and regression losses. What sets us apart from other trackers is that our classification loss incorporates the central loss function, eliminating the need for separate listing.

**Figure 3:**
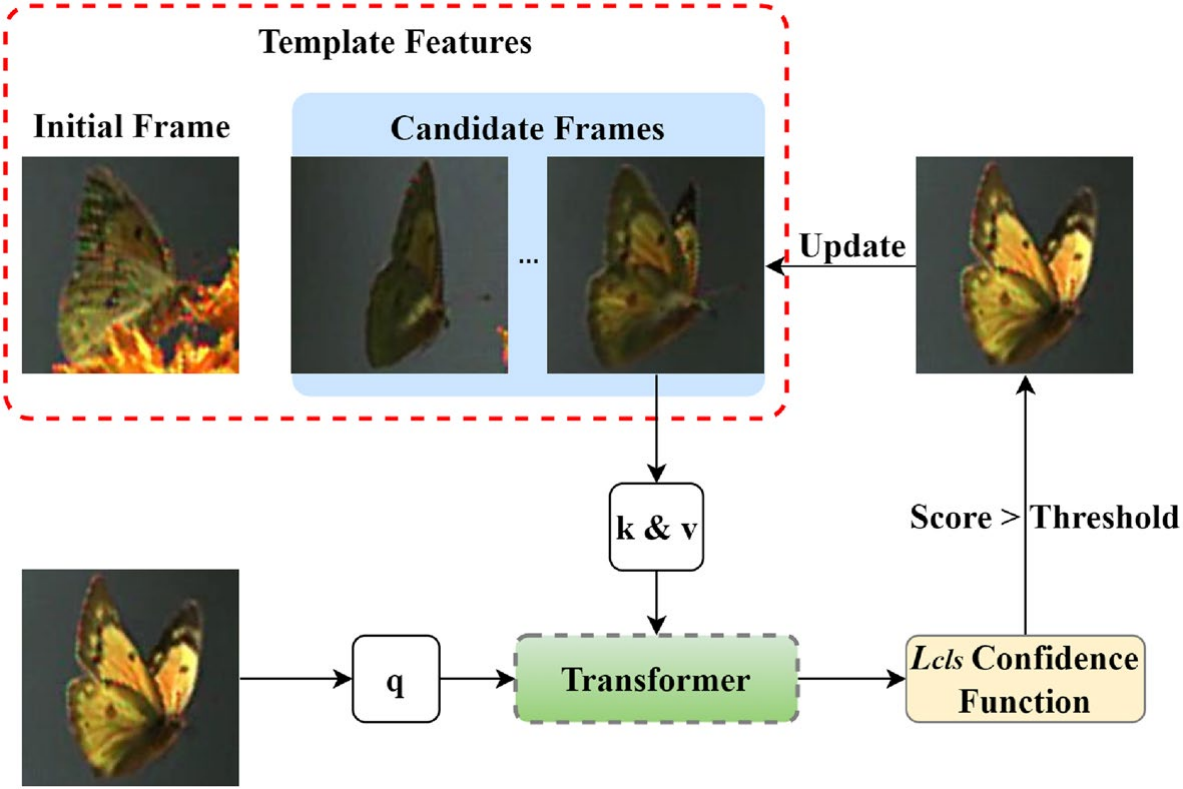
DTU: Selecting reliable latest frames from historical frames using the confidence function of the classifier.

**Dynamic template updater (DTU)** Long-term complex tracking tasks involve appearance deformations of the target, thereby rendering conventional tracking templates ineffective. Enhancing the robustness of online templates is crucial for the accuracy of tracking tasks. Drawing inspiration from pioneering methods like STARK^11^ and NeighborTrack^18^, we have crafted a dynamic template updating strategy, as depicted in Fig. 3, that incorporates attention mechanisms and multi-layer perceptrons. This approach filters out reliable recent frames from historical data. By comparing the initial frame with potential tracking frames, we identify the most recent frame with a target classifier confidence exceeding a set threshold and subsequently update the dynamic template accordingly. The target classifier leverages a cross-entropy loss function, expressed as:

11 $$\begin{aligned} {{L}_{cl\text {s}}}={{y}_{i}}\log ({{p}_{i}})+(1-{{y}_{i}})\log (1-{{p}_{i}}). \end{aligned}$$

Where ${{y}_{i}}$ signifies the ground truth label, and ${{p}_{i}}$ denotes the predicted confidence level.

### Ethical approval

This article does not contain any studies with human participants or animals performed by any of the authors. No humans or any individual participants are involved in this study.

## Experiments and discussion

### Implementation details

**Training phase** During training, we utilized LaSOT^23^, TrackingNet^24^, GOT-10k^1^ and COCO 2017^25^ datasets, totaling 40,000 samples per epoch. We employed the GOT-10k validation set, comprising 10,000 samples per 5 epochs, for validation. Our entire network underwent training, enhanced by random image flipping and color jittering to diversify samples. We randomly selected two training frames and one testing frame from video sequences to form training sub-sequences, ensuring frames had identical resolutions and bounding boxes. Target information was encoded using both training frame states and supervised by testing frame bounding boxes. Unlike trackers with Swin-transformer-tiny, we reduced its output dimension to 256 and the Linear layer’s dimension to 1024, resulting in a network with 21.9M parameters and 24.5G FLOPs. We trained with two 4080 GPUs, AdamW optimizer, 300 epochs, a learning rate of 0.00015, decaying by 0.2 at epochs 150 and 250.

**Online testing phase** During tracking, we employed an NVIDIA RTX 2080Ti GPU. By maintaining historical frames as candidates, we selected recent frames with high target classifier confidence. Leveraging annotated initial frames and neighboring data, we mitigated similar target interferences. The target’s central position was determined via the target model’s score map, and dense bounding box predictions refined the final target box.

### Comparison with the previous methods

#### Comparison with the state-of-the-art trackers

To validate our tracker’s efficacy, we conducted experiments on five benchmark datasets: GOT-10k, LaSOT, LaSOTExt^26^, TrackingNet and OTB100^27^, Our tracker outperforms state-of-the-art trackers on these challenges.

**Figure 4:**
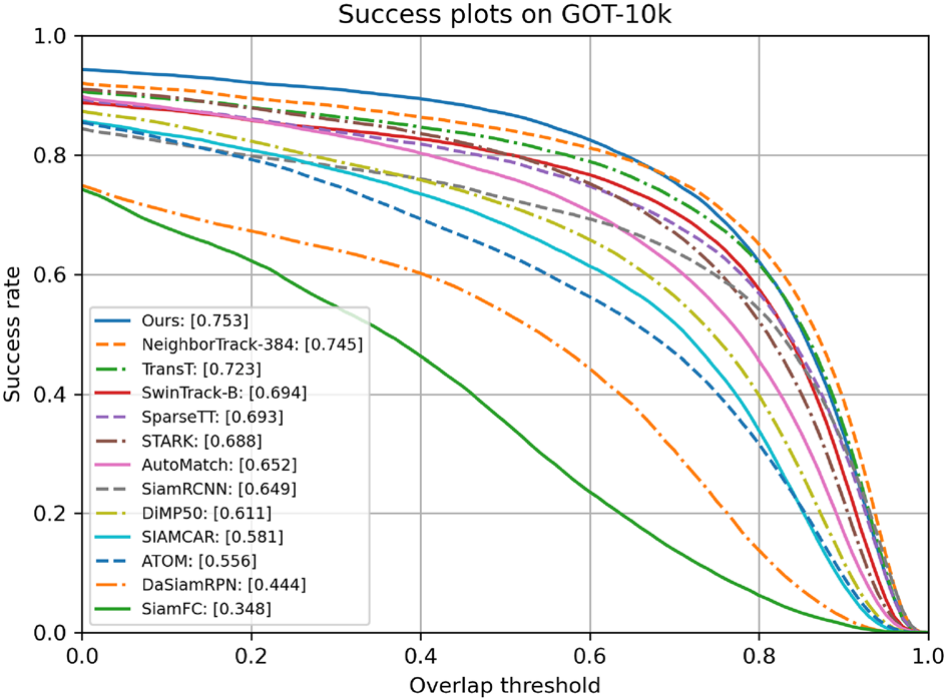
Comparison with state-of-the-art trackers on GOT-10k.

**Figure 5:**
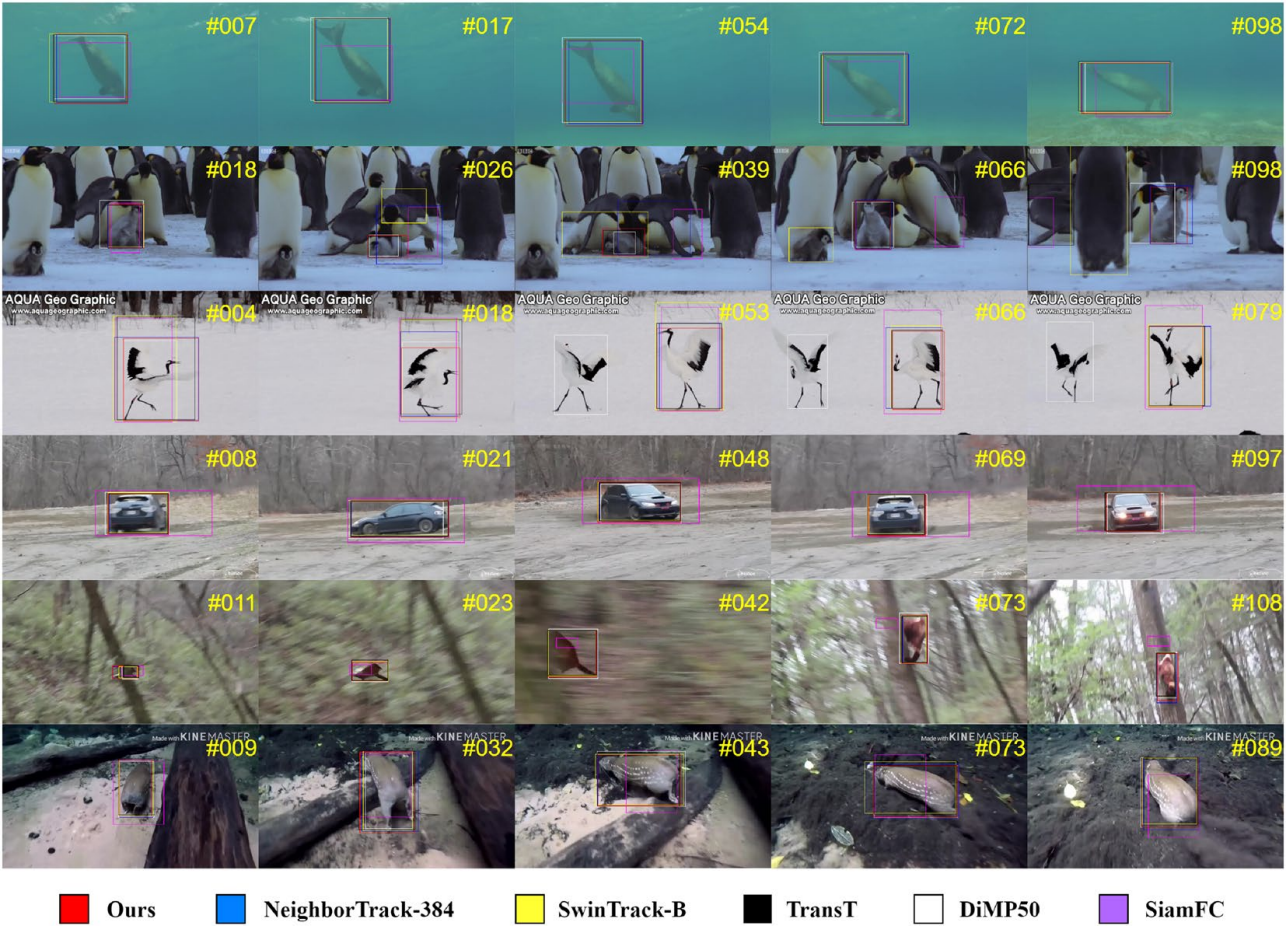
ASACTT is compared with other trackers in terms of tracking prediction boxes across 6 video sequences from the GOT-10K dataset(Test-00001, Test-0000006, Test-000012, Test-000023, Test-000066, Test-00099 ).

**GOT-10k** The GOT-10k dataset, comprising 180 non-overlapping testing video sequences, is used for tracking evaluations. Test results are officially submitted for assessment. Figure 4 shows that ASACTT outperforms top trackers like NeighborTrack-384^18^, TransT^4^, SwinTrack-B^12^, SparseTT^8^, START^11^, AutoMach^28^, SiamRCNN^20^, DiMP50^29^, SIAMCAR^30^, ATOM^19^, DaSiamRPN^31^ and SiamFC^2^, achieving state-of-the-art results on the GOT-10k benchmark. Figure 5 visualizes the performance of various trackers on the GOT-10K dataset, highlighting ASACTT’s effectiveness in handling complex scenarios, including fast motion, background noise, occlusions, deformations, scale variations, and lighting changes, compared to other methods like TransT, NeighborTrack-384, SwinTrack-B, DiMP50, and SiamFC, evident in its precise tracking predictions.

Despite using Transformer structures, our ASACTT tracker outperforms NeighborTrack-384, TransT, SwinTrack-B, SparseTT, and START. ASACTT’s unique ASA Transformer structure captures superior global information and focuses on target-relevant features, resulting in robust dynamic template updater and exceptional tracking performance.

**LaSOT** The LaSOT dataset is a large-scale long-term tracking dataset, with its test set comprising 280 video sequences. According to its evaluation protocol, we conducted comparative experiments involving our tracker ASACTT and 13 other trackers: SparseTT, STMTrack^22^, TransT, MDNet^32^, Ocean^33^, DiMP, DaSiamRPN, SiamMask^34^, GlobalTrack^35^, ATOM, SiamRPN++, ECO and SiamFC.

**Figure 6:**
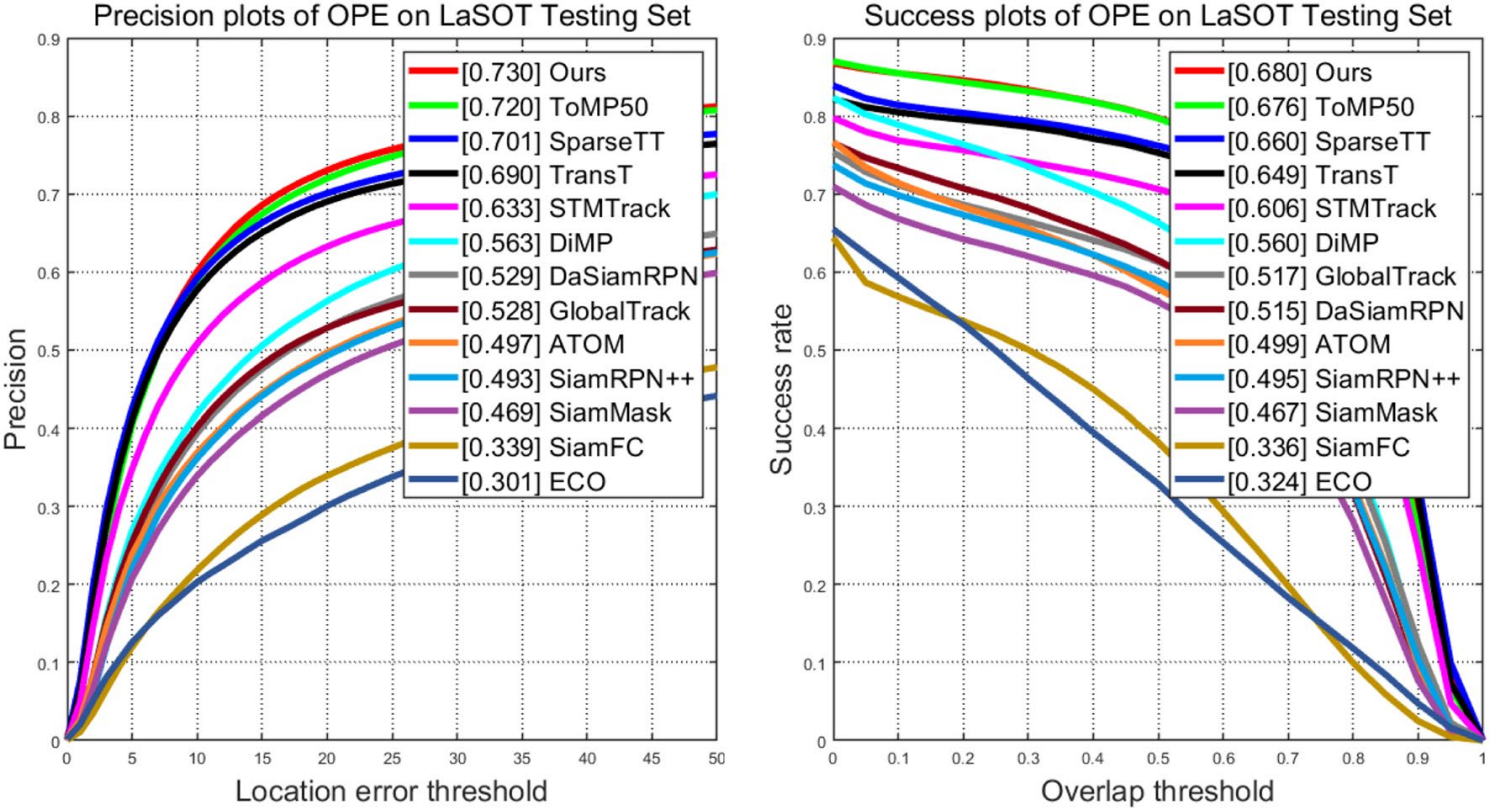
The success rate and accuracy rate of LaSOT.

Figure 6 assesses 14 challenging attributes(aspect ratio changes, background clutter, camera motion, deformation, fast motion, complete occlusion, illumination changes, low resolution, motion blur, out-of-view, partial occlusion, rotation, scale changes, viewpoint changes), revealing that our ASACTT tracker achieves 68.04% accuracy, surpassing ToMP50, SparseTT, and TransT by 0.4%, 2%, and 3% respectively. In precision, it reaches 73%, topping ToMP50 and SparseTT by 1% and 3%. Top performers, including ASACTT, utilize the Transformer structure, confirming its advantage over CNNs. ASACTT and SparseTT employ Swin-Transformer, a backbone that offers superior feature representation. Despite both ASACTT and SparseTT employ Swin-Transformer, a backbone that offers superior feature representation, our ASACTT stands apart. It addresses the challenges of a fixed sparse attention threshold and limited scalability, introducing a DTU, enhancing robustness and delivering outstanding performance.

**Figure 7:**
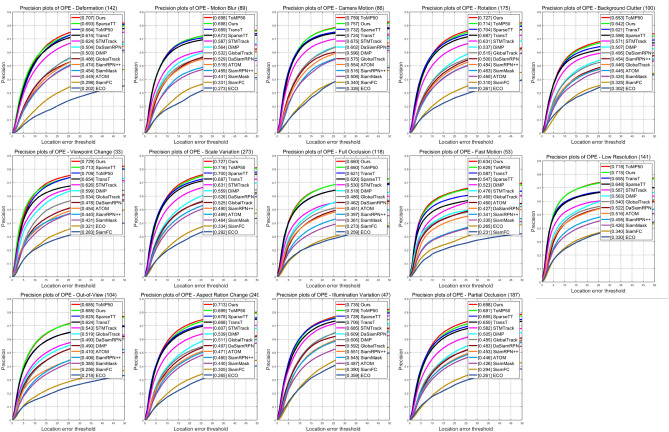
The tracking results of 13 trackers on 14 different attributes in terms of accuracy.

**Figure 8:**
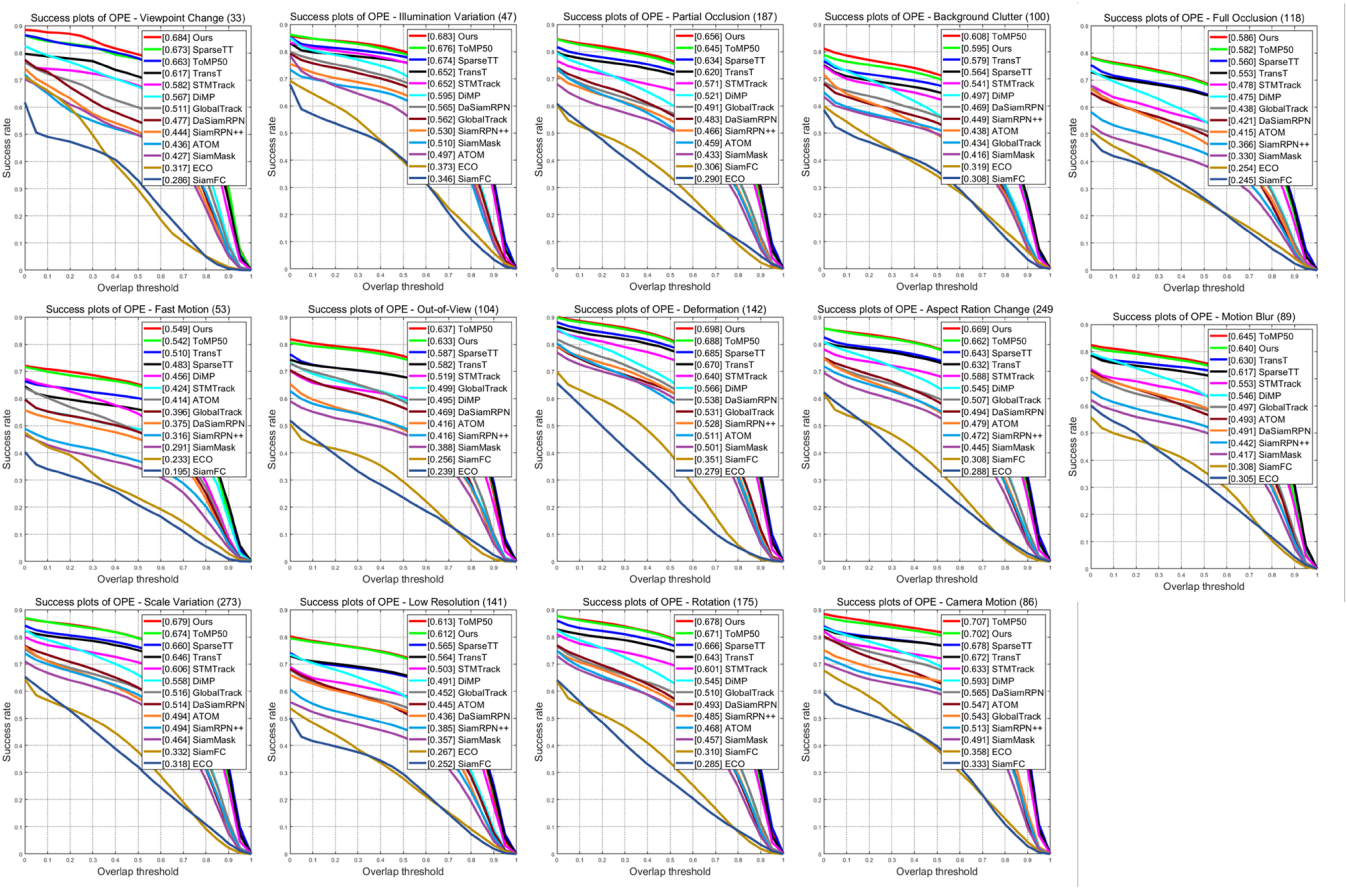
The tracking results of 13 trackers on 14 different attributes in terms of success.

Figures 7 and 8 showcase the performance of our ASACTT method versus 13 trackers across 14 challenging attributes. Our approach, integrating ASA and MCA Transformers with a DTU, excels in most attributes, particularly in tackling challenges like lighting changes, occlusions, deformations, and rapid motion. ASA compensates for MCA’s local target information gap, while the Swin-Transformer’s robust features enhance information capture from all search regions. The dynamic template updater, leveraging depth maps, positional data, and target state updates, filters templates through classification losses, optimizing template quality, robustness, and prediction accuracy for the next frame.

Although ASACTT lags behind ToMP50 in areas like deformation, camera motion, background clutter, out-of-view, and low resolution, we delved into the matter. Our Swin-Transformer-based approach boasts superior global vision and feature representation, surpassing ResNet. However, in deformations and camera motion, Swin-Transformer’s self-attention mechanism, which hinges on input sequences, may falter with significant target deformations or camera-induced positional shifts, impacting tracking performance. Conversely, ToMP50’s ResNet backbone adeptly captures local image details, adapting well to deformations and camera movements.

**Figure 9:**
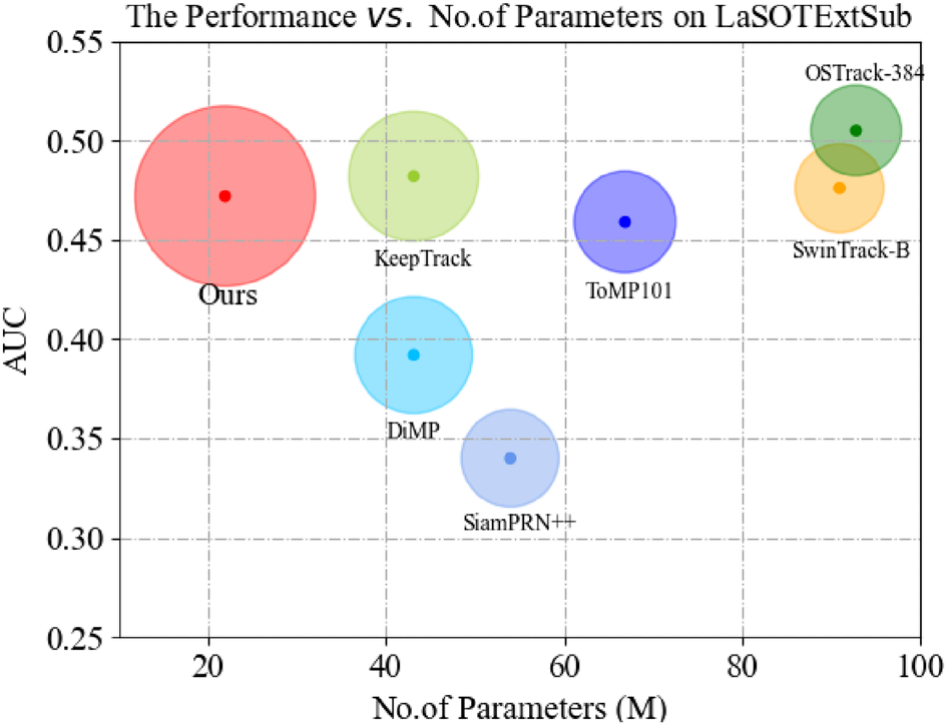
Comparison with state-of-the-art trackers on LaSOTExt using success (AUC) score and No. of Parameters (M).

**LaSOTExt** The LaSOTExt dataset augments LaSOT with 150 videos from 15 new categories, encompassing complex scenarios like similar, small, and fast-moving targets. Our ASACTT tracker boasts a 47.2% accuracy, surpassing most rivals. As shown in Fig. 9, ASACTT outperforms ToMP-101 by 1.3% in accuracy despite its smaller parameter size. However, it lags behind SwinTrack-B, KeepTrack, and OSTrack-384 by 0.4%, 1%, and 3.3% respectively. Notably, KeepTrack’s parameters are twice as large, while SwinTrack-B and OSTrack-384 exceed it fourfold. This is due to ASACTT’s lack of additional parameters for target information collection.

**TrackingNet** The TrackingNet dataset, encompassing diverse objects and tracking scenarios, consists of 511 test video sequences. We rigorously evaluated our ASACTT method based on its protocol, achieving a success rate of 82.3%, precision normalized at 87.1%, and precision at 80.3% (Table 1). These figures outperform most methods, trailing only 0.2% behind SwinTrack-B, a model with a larger parameter capacity that captures more target features. Our ASACTT, integrated with Swin-Transformer, ASA and DTU, exhibits strong global search, feature representation, and robustness to deformations and similar-object interference. Despite its compactness, the model thrives in large-scale, multi-class datasets.

**Table 1:** Performance comparison on TrackingNet.

Method	AUC (%)	Precision (%)	Norm. Prec (%)
SiamFC^2^	57.1	66.3	53.3
DiMP50^29^	74.0	80.1	68.7
SiamR-CNN^20^	81.2	85.4	80.0
TrDiMP^5^	78.4	83.3	73.1
TransT^4^	81.4	86.7	80.3
AutoMatch^28^	76.0	–	72.6
STARK^11^	82.0	86.9	-
SwinTrackB^12^	82.5	87.0	80.4
SwinTrack-T^12^	80.8	77.9	85.5
Ours	82.3	87.1	80.3

**Figure 10:**
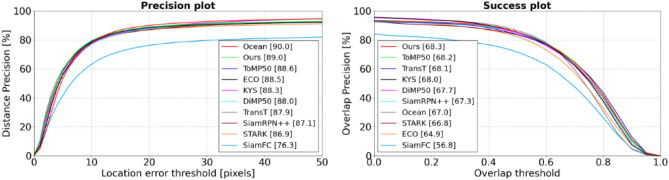
The success rate and accuracy rate of OTB100.

**OTB100** We conducted comparative experiments on the OTB100 dataset, benchmarking our tracker against leading methods like TransT, Ocean, SiamRPN++, ECO, ToMP50, KYS^36^, DiMP50, STARK, and SiamFC. As shown in Fig. 10, ASACTT achieved the top success rate, narrowly trailing Ocean in accuracy. This hints at potential improvements in boundary prediction box estimation.

#### Comparison with efficient trackers

To further validate the performance of our tracker, ASACTT, we have conducted a comparative analysis on the LaSOT dataset, evaluating the AUC, precision, normalized precision, FLOPs, and speed of several recent Transformer-based efficient trackers, including MixFormer, JointNLT, GTELT, EMAT, and ASAFormer. As illustrated in Table 2, ASACTT stands out as the leader in precision, surpassing ASAFormer and EMAT by 0.3% and 3.4% respectively.

**Table 2:** AUC, precision, normalized precision, Flops and on LaSOT and the speed of different trackers. All speeds are reported on the RTX 2080Ti GPU other than those denoted by *.

	MixFormer-1K	JointNLT	GTELT	EMAT	ASAFormer	Ours
	^37^	^38^	^39^	^40^	^41^	
AUC.	67.9	60.4	67.7	64.6	67.7	68.0
Pre.	73.9	63.6	73.2	67.0	73.2	73.0
nPre	77.3	–	–	73.7	–	77.8
FLOPs	23G	42.0G	–	–	24.15G	24.5
FPS	25	39*	26	35	16.3*	36

### Ablation study

To assess the contribution of our tracker’s modules, we performed ablation experiments on the got10k dataset, evaluating their effectiveness through success rate metrics.

**The impact of each component on our tracker.** Table 3 reveals that our tracker, ASACTT, achieves the lowest success rate of 71.7% when deprived of position encoding. Without this encoding, the tracker fails to pinpoint the target accurately, risking tracking failures. Similarly, excluding DTU drops the success rate to 72.9%, emphasizing the criticality of real-time updates and an optimized template library. Furthermore, disregarding ASA diminishes the results by 0.8%, underscoring its effectiveness in focusing on locally pertinent target information.

**Table 3:** Ablation studies on ASACTT.

Modification	GOT-10k Success (%)
Ours	75.3
No Pos.	71.7
No DTU	72.9
No ASA	74.5

**The impact of different backbones on our tracker.** As shown in Table 4, to demonstrate the superiority of various backbones for our tracker, we kept other components constant and replaced them with ResNet50 and ResNet101. Their success rates were 71.8% and 73.2%, respectively. This is attributed to the fact that the SwinTransformer adopts a hierarchical architecture design similar to convolutional networks. As the network depth increases, the resolution of feature maps gradually decreases, enabling better capture of target features at different scales. Additionally, the design of shifted windows enhances the model’s spatial relationship modeling capability across windows. This, in turn, improves the tracker’s ability to handle challenging issues such as target occlusion and deformation.

**Table 4:** Effects of different backbones on tracker.

Backbone	GOT-10k SUC(%)	Parameters (M)	FLOPs(G)
ResNet50	71.8	26.1	25.7
ResNet101	73.2	45.1	44.2
SwinTransformer-Tiny(256)	75.3	21.9	24.5

**The impact of different attention mechanisms on our tracker.** As shown in Table 5, our tracker ASACTT, which employs ASA instead of multi-head self-attention(MSA), not only exhibits enhanced tracking accuracy but also boasts improved tracking speed. Furthermore, when we substituted our ASA with conventional sparse attention(SA), we discovered that ASA not only maintained tracking speed but also genuinely augmented tracking performance, thereby validating the efficacy of the ASA module.

**Table 5:** Effects of different attentions on tracker.

Attention	GOT-10k SUC (%)	FPS
MSA+MCA	74.5	33
SA+MCA	74.9	36
ASA+MCA	75.3	36

## Conclusion

In this paper, we propose a novel Siamese network architecture called ASACTT. This architecture utilizes Swin Transform Tiny as its backbone, significantly bolstering the global feature representation capabilities. Furthermore, ASACTT dynamically adjusts the sparse attention threshold based on the length and content of the input image sequence, optimizing the coordination of sparse attention mechanisms. Additionally, during the online tracking stage, by integrating position encoding and historical candidate data through a DTU module, ASACTT dynamically updates the optimal template frame. This approach minimizes template errors and subsequently enhances the robustness of the tracker. Comprehensive evaluations performed on challenging datasets have unequivocally showcased the superiority of ASACTT over other mainstream trackers, particularly those with comparable model parameters.

## Data Availability

The data that support the findings of this study are available from the corresponding author upon reasonable request.
